# Improving physical activity after stroke via treadmill training and self management (*IMPACT*): a protocol for a randomised controlled trial

**DOI:** 10.1186/s12883-018-1015-6

**Published:** 2018-01-30

**Authors:** Sandra G. Brauer, Suzanne S. Kuys, Jennifer D. Paratz, Louise Ada

**Affiliations:** 1 Discipline of Physiotherapy, School of Health and Rehabilitation Sciences, The University of Queensland, St Lucia, QLD Australia; 2 School of Physiotherapy, Faculty of Health Sciences, Australian Catholic University, Banyo, QLD Australia; 3 School of Allied Health Sciences, Griffith University, Southport, QLD Australia; 4 Discipline of Physiotherapy, Faculty of Health Sciences, The University of Sydney, Sydney, NSW Australia

**Keywords:** Stroke, Randomised, Treadmill, Walking, Cardiorespiratory fitness, Physical activity

## Abstract

**Background:**

The level of physical activity undertaken by stroke survivors living in the community is generally low. The main objectives of the *IMPACT* trial are to determine, in individuals undergoing rehabilitation after stroke, if 8 weeks of high-intensity treadmill training embedded in self-management education (i) results in more physical activity than usual physiotherapy gait training and (ii) is more effective at increasing walking ability, cardiorespiratory fitness, self-efficacy, perception of physical activity, participation, and health-related quality of life as well as decreasing cardiovascular risk, and depression, at 8 and 26 weeks.

**Methods:**

A prospective, two-arm, parallel-group, randomised trial with concealed allocation, blinded measurement and intention-to-treat analysis, will be conducted. 128 stroke survivors undergoing rehabilitation who are able to walk independently will be recruited and randomly allocated to either the experimental or control group, who will both undergo gait training for 30 min, three times a week for 8 weeks under the supervision of a physiotherapist. Outcomes will be measured at baseline (Week 0), on completion of the intervention (Week 8) and beyond the intervention (Week 26). This study has obtained ethical approval from the relevant Human Research Ethics Committees.

**Discussion:**

Improving stroke survivors’ walking ability and cardiorespiratory fitness is likely to increase their levels of physical activity. Furthermore, if education in self-management results in sustained high levels of physical activity, this should result in improved participation and quality of life.

**Trial registration:**

This trial was registered with the Australian New Zealand Clinical Trials Registry (ACTRN12613000744752) on 4th July, 2013.

## Background

Stroke survivors have three times the risk of further cardiovascular events than the general population [1, 2]. There is strong evidence that physical activity has a protective effect against stroke [3, 4], it can reduce cardiovascular risk factors in people with stroke [5] and not undertaking enough physical activity increases the risk of recurrent stroke [6, 7]. The level of physical activity undertaken by stroke survivors living in the community is generally low [8, 9]; less than half that of age-matched adults [10, 11]. However, increasing the level of physical activity after stroke is challenging, perhaps because multiple factors contribute. For example, lack of physical activity is correlated with poor cardiorespiratory fitness [12], reduced walking ability [13] and low self-efficacy [14].

High-intensity treadmill training, i.e., training to a target heart rate around 40–60% of heart rate reserve, has been shown to increase cardiorespiratory fitness in people with chronic stroke [15, 16]. However, this type of training is not usually started during inpatient rehabilitation. In a pilot study, we have shown that high-intensity treadmill training during inpatient rehabilitation is feasible, is not detrimental to the walking pattern and may result in an improvement in walking ability [17]. However, we have not shown that it increases cardiorespiratory fitness.

Furthermore, one of the main findings of studies investigating interventions to improve cardiorespiratory fitness after stroke, is that the benefit is only partially maintained beyond the period of intervention [15]. It is therefore important to support stroke survivors to exercise independently in the long term [18]. A self-management approach to rehabilitation has been recommended as a strategy to optimise outcomes for stroke survivors after discharge from hospital [19]. Interventions that are grounded in health behaviour theory, and which target psychological constructs with evidence based intervention activities, are more successful than those which are neither theory- nor evidence-based [20]. The Health Action Process Approach (HAPA) has been found to be useful, particularly in the rehabilitation setting, for predicting and explaining changes in health behavior [20]. It consists of two phases – a motivational phase and a volitional phase. In the motivational phase, the perception that a person is at risk, their positive outcome expectancies for completing a certain behaviour, and their perceived self-efficacy for performing a certain action interact to drive the formation of a behaviour intention [20]. In the volitional phase, self-regulatory skills and strategies (action planning, coping planning, recovery self-efficacy and social support) are employed to translate intentions into action [20]. Intervention behaviour change techniques can be tailored to participants dependent on their phase. The validity of the HAPA has been tested in the intervention context with people in the rehabilitation setting, including individuals with stroke [21, 22] and could be useful to address the behavior change issue of modifying physical activity after stroke. To augment this approach, goal setting theory can be employed to target goal difficulty and specificity [23].

It appears that a comprehensive, patient-centered, goal-oriented approach to lifestyle modification is required early in rehabilitation that addresses both the ability for stroke survivors to be active, and the motivation to do so. Maintaining high levels of physical activity via improved walking ability and ongoing cardiorespiratory fitness should also confer a range of benefits, such as an improvement in self-efficacy, participation, and health-related quality of life and a decrease in cardiovascular risk [24], and depression. If a sufficient training level can be reached, there is likely to be an impact on lipid profile: high sensitivity C- reactive protein (HSCRP), total cholesterol (TC), triglycerides (TRG) high density and lipoprotein cholesterol (HDL-C) and low density lipoprotein cholesterol (LDL-C). The link between modification of the lipid profile and inflammatory biomarkers to carotid intima–media thickness and subsequent cerebro and cardiovascular events has been convincingly demonstrated [25]. Additionally, there is strong evidence that moderate intensity exercise can improve lipid profiles and inflammatory markers, including highly sensitive C-reactive protein (HSCRP), an independent predictor of cardiovascular events in both primary and secondary prevention [26–29]. However, until recently it has been difficult to exercise stroke patients (who due to their pathophysiology are already at risk of future cardiovascular events), at a sufficient level to achieve these benefits. A recent study [30] has provided the first evidence that high-intensity treadmill training in chronic stroke survivors reduces cardiovascular risk markers (cerebral vasomotor reactivity), implying that this form of exercise intervention may confer a neuroprotective effect. High-intensity treadmill training may lower cardiovascular disease risk factors for stroke survivors which in turn should result in secondary prevention of future cardiovascular and cerebrovascular events.

We are therefore conducting a randomized trial investigating the effect of high-intensity treadmill training during inpatient rehabilitation embedded in self-management. This will be compared with usual physiotherapy gait training to ensure a similar volume of training. The main objectives of the *IMPACT* trial are to determine, in individuals undergoing rehabilitation after stroke, if 8 weeks of high-intensity treadmill training embedded in self-management:

(i) results in more physical activity than usual gait training; and
(ii) is more effective at increasing walking ability, cardiorespiratory fitness, self-efficacy, perception of physical activity, participation, and quality of life as well as decreasing cardiovascular risk, and depression; immediately after the intervention at 8 weeks and beyond the intervention at 6 months.

## Methods

**Fig. 1:**
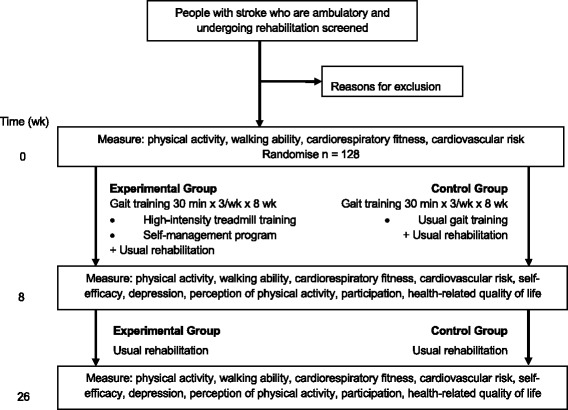
Design of the study

A prospective, two-arm, parallel-group, randomised trial with concealed allocation, blinded measurement and intention-to-treat analysis will be undertaken with stroke survivors undergoing rehabilitation (Fig. 1). Participants will be recruited from rehabilitation units in two states (Queensland and NSW) in Australia. A list of centres is available on the trial registry. Participants will be randomly allocated to two groups. Gait training for the experimental group will be high-intensity treadmill training plus self management and for the control group will be usual gait training. Both groups will have the same amount of time spent on gait training. To minimise the risk of contamination, the experimental group will receive their intervention from a different therapist in a different location from the control group. The rest of the multi-disciplinary rehabilitation program will be unchanged. The end point of the intervention phase of the study will be 8 weeks and the end point of the follow-up phase will be 26 weeks. Outcomes will be measured at baseline (Week 0), on completion of the intervention (Week 8) and beyond the intervention (Week 26). Both data collection and data analysis will be completed by researchers who are blind to group allocation. Participants will be asked not to reveal the nature of their intervention to measurers. It is not possible to blind participants or therapists to group allocation. The study protocol has been approved by the appropriate Human Research Ethics Committees and has been registered on the Australian New Zealand Clinical Trials Registry.

### Participants, therapists, centres

Individuals with stroke will be screened and invited to participate if they are: within two months of a stroke (confirmed by CT scan or clinical diagnosis); aged 18 years or older; able to walk 10 m independently (without physical assistance but with or without an aid); and able to follow three-stage commands. They will be excluded if they: were unable to walk independently prior to current stroke; have co-morbidities relating to the lower limbs that might limit their walking (such as arthritis); have unstable cardiac status (which would preclude them from participating in intensive training); unable to return for assessment or training; or are unable to provide informed consent. Information that will be collected to describe the sample will include: age, sex, description of the stroke (time, side affected, type, location, severity), aphasia, recombinant tissue plasminogen activator (rt-PA), medications, co-morbidities, and previous living arrangements.

Therapists delivering the intervention will be included if they are physiotherapists with more than 5 years clinical experience, or experience in treadmill training or self-management.

Centres will be included if they contain a rehabilitation unit that has a throughput of stroke of more than 50 per year.

### Randomisation

Randomisation will be computer-generated, independent and concealed. The allocation sequence has been computer-generated by an independent researcher not involved in this study. For each centre, allocation to the experimental or control group will occur in random permuted blocks, so that after every block (of 4–6 participants), the experimental and control group will contain equal numbers [31]. Group allocation is stored in numbered, opaque envelopes in order to provide secure randomisation. After baseline measures have been collected, the therapist providing the intervention opens the envelope and reveals the group allocation. In this way, the allocation sequence is concealed from on-site recruiters.

### Intervention

Both the experimental and the control group will undergo gait training during rehabilitation as part of this trial for 30 min, three times a week for 8 weeks under the supervision of a therapist. Gait training for the other part of the week will be as usual. The gait training may be carried out across inpatient and outpatient departments – since some participants may be discharged home before the end of the 8-week training. No other part of the multidisciplinary rehabilitation program will be controlled. Randomisation should ensure that any effect of other interventions will be the same for both groups, therefore, other rehabilitation will not be withheld. Typically for stroke patients, rehabilitation broadly consists of as much physical practice as possible including early mobilisation and upper limb training as specified by the *Clinical Guidelines for Stroke Management 2010* [32].

#### Experimental group

The experimental group will receive high-intensity treadmill training and self-management education. The high-intensity treadmill training will be based on our pilot program [17] of up to 30 min walking on a treadmill at an intensity of 40–60% heart rate reserve or a Borg Rating of Perceived Exertion [33] of 11–14; the minimum required for training cardiorespiratory fitness [34]. Target heart rates will be calculated according to Karvonen method [34] adjusting for beta-blockers for those taking heart rate lowering medication. Participants will start at an intensity of 40% heart rate reserve, progressing each week by a 5–10% increase until 60% heart rate reserve is reached. Heart rate will be recorded via a portable heart rate monitor. While on the treadmill, participants will be encouraged to hold the handrail and a physiotherapist will provide assistance as required to ensure foot clearance during swing phase. In addition, an assistant will be available to operate the controls and the safety stop cord will be attached to participants at all times.

The self-management education in this intervention is underpinned by two theoretical models: the Health Action Process Approach (HAPA) [35] and Goal Setting Theory [36]. At the beginning of the study, participants in the treadmill training group will receive a detailed workbook to complement the treadmill training component of the intervention. The workbook will serve a dual purpose of encouraging self-monitoring of physical activity and guiding participants through the process of setting short- and long-term goals as well as formulating action plans and coping strategies. The therapist delivering the intervention will allocate a proportion of each treadmill session to addressing the self-management-related tasks outlined in the workbook. The multi-modal behaviour change techniques to be employed have been selected to align with the theoretical basis of the intervention and progress over time. These include: education, behavioural instruction, self-monitoring, goal setting and goal review, feedback, problem solving, action planning and coping planning. The way in which these behaviour changes strategies are operationalized is described below. In order to address risk perception and outcome expectancies, participants will be informed about the consequences of their action (inaction) and asked to consider the costs and benefits of their behaviour. Task self-efficacy (for treadmill walking and physical activity generally) will be enhanced by providing instruction on how to perform the behaviour, prompting of self-monitoring (using the workbook), and the provision of feedback on performance. Participants will be asked to set specific, relevant and patient-centred short- medium- and long-term goals as a way of fostering intention formation. Action planning and coping planning (i.e., problem solving and identifying strategies to overcoming potential barriers to achieving behaviour change goals) will be used to build self-efficacy for behaviour maintenance. As participants reach the end of the treadmill intervention, and transition to independent physical activity practice, further strategies to encourage maintenance self-efficacy will be employed. These will include discussions to prompt participants to review their behavioural goals and gather information on alternative options to incorporate sufficient physical activity program in their life.

Physiotherapists delivering the experimental intervention will be trained in high-intensity treadmill training and self-management education and provided with guidelines, training manuals and support. Information describing the high-intensity treadmill training (e.g., duration, distance walked, observed heart rate, perceived exertion, adverse events) and self-management education (e.g. frequency of participant-initiated conversations on coping planning) will be recorded to be able to describe the intervention accurately. Adherence to the experimental intervention will be monitored by reviewing the recording sheets and by auditing 1–2 sessions per therapist.

#### Control group

The control group will receive usual gait training delivered by the usual treating physiotherapist. Usual gait training will not be prescribed, but quantified (e.g., time and distance walked, tasks practiced will be recorded) to be able to describe the intervention accurately. The recordings of usual gait training at the beginning and the end of the study end will be examined to check for contamination from the experimental intervention.

### Measurement

Since the main aim the study is to improve physical activity, the primary outcome will be physical activity measured as steps/day over four days at Weeks 0, 8, 26. Secondary measures collected at Weeks 0, 8 and 26 will include walking ability, cardiorespiratory fitness and cardiovascular risk. Secondary measures collected at Weeks 8 and 26 will include self-efficacy, depression, perception of physical activity, participation, and health-related quality of life.

#### Primary

- Physical activity will be measured over 4 days using a triaxial accelerometer (activPAL3™, PAL Technologies Ltd., Glasgow, UK) secured to the middle of the anterior non-paretic thigh and reported as steps/day. This device has been shown to demonstrate concurrent validity for step count, by demonstrating low error (1.6%) and high concurrence with direction observation (ICC 0.99, 95% CI 0.98 to 1.0) in people with stroke walking in an outdoor circuit [37], and test-retest reliability when walking indoors across days (ICC 0.98, 95% CI 0.93–0.99) [37].

#### Secondary

- Walking ability will be measured using the 6-min Walk Test and the 10-m Walk Test. The 6-min Walk Test will be conducted according to the American Thoracic Society guidelines [38] and reported as distance walked in m. It is commonly used as a functional submaximal measure of oxygen uptake and exercise capacity [39] and is a valid and reliable clinical measure of walking ability in stroke survivors [40]. The 10-m Walk Test will be conducted at preferred and fast speeds over a 15 m course to remove any acceleration and deceleration, using usual gait aids, and reported as mean (SD) speed in m/s.
- Cardiorespiratory fitness will be measured from the 6-min Walk Test and reported as VO_2_Peak in ml/kg/min [41]. Heart rate, blood pressure, rate pressure product (systolic arterial pressure x heart rate) and VO_2_Peak will be recorded at rest and during the 6-min Walk Test, by a fully portable compact metabolic system using breath by breath analysis (Metamax 3B, Cortex, Biophysik, Germany) shown to be stable over time and accurate when tested against a gold standard criterion [42]. Test-retest reliability of cardiorespiratory variables measured using the Metamax 3B has been demonstrated for people with stroke when completing the 6-min Walk Test (ICCs range 0.76–0.97, error < 4%) [43].
- Cardiovascular risk will be measured from lipid profile and inflammatory markers. A finger stick will be taken and immediately analysed on a point of care device (Cholestech LDX, Alere™, Brisbane) [44] for lipid profile: total cholesterol (TC), low density lipoprotein (LDL), high density lipoprotein (HDL), ratio of total cholesterol to HDL (TC/HDL) and triglycerides (TRG). This instrument has been shown to have excellent validity compared to gold standard laboratory analysis [44]. Lipid profile measures for the Cholestech LDX had ICCs ranging from 0.87–0.99. A blood sample will be taken and analysed for inflammatory markers (high sensitivity CRP) [25].
- Self-efficacy of walking ability will be measured using the Ambulatory Self-Confidence Questionnaire, which has demonstrated reliability and validity in the older population [45]. The 22 items will be reported as a score from 0 to 10 where 0 is no confidence.
- Depression will be measured using the Hospital and Anxiety Depression Scale [46] and reported as a score from 0 to 21 on the depression subscale, where 0 is no depression. It has established validity in people with stroke [47].
- Perception of physical activity will be measured using the Physical Activity Scale for Individuals with Physical Disabilities in the domains of home repair, lawn and garden work, housework, vigorous sport and recreation, moderate sport and recreation, and occupation and transportation and the 13 items reported as a score from 0 to 100 MET hr./day, where 0 is no activity [48]. This survey has demonstrated criterion validity and test-retest reliability [49].
- Participation will be measured using the Impact on Participation and Autonomy Questionnaire [50] in the domains of indoor and outdoor autonomy, family role, social relations, work and education and the 31 items reported as a score from 30 to 155, where 30 is very little impact. It has demonstrated validity and test-retest reliability (ICC 0.83 to 0.91) in people with chronic disorders [51].
- Health-related quality of life will be measured using the EuroQual-5D [52] in the domains of mobility, usual activities, personal care, pain/discomfort and anxiety/depression as well as a measure of overall health state using a visual analogue scale. The domains will be reported as a utility score between 0 and 1, and the overall health state reported between 0 and 100, where 0 is poor health. It has established psychometric properties in people with stroke [53].

Data quality will be maintained by assessors being experienced physiotherapists trained in the collection of outcome measures. Double data entry will be performed separately by two research assistants blind to group allocation to reduce the potential for transcription error. All data will be de-identified to maintain confidentiality during and after the trial. Participants will receive a summary of results at the completion of the trial.

### Sample size

One hundred twenty eight participants will be recruited. The sample size has been calculated to be able to detect an effect size of 1200 steps/day with 80% power at a two-tailed significance level of 0.05. This between-group difference was based on an estimate that participants would take 4000 (SD 2000) steps/day at baseline and that the control intervention would increase this to 5200 step/day and the experimental intervention would increase this to 6400 steps/day, putting them above a sedentary level for older adults [54]. The smallest number of participants to detect a difference in physical activity of 1200 steps/day between two groups estimated from independent samples is 55 participants per group. On the assumption from previous trials with this population that approximately 15% of the participants may be lost to follow up during the course of the study, we have set a target of 128 participants in total. In order to reach the target sample size, the trial will be carried out across multiple centres, and a researcher will be employed to screen all stroke survivors at each centre.

### Statistical analysis

Primary and secondary outcomes including physical activity, walking ability, cardiorespiratory fitness, cardiovascular risk, self-efficacy, perception of physical activity, participation, and quality of life will be reported as point estimates and measures of variability. An intention-to-treat between-group analysis will be performed on all outcome measures. Between-group differences will be analysed using generalized mixed effects models for continuous data, since this analysis does not require casewise deletion due to missing data. Data will be presented as mean difference (95% CI). Baseline characteristics judged to be clinically different between groups may be included as covariates.

## Discussion

This study has the potential to reduce disability and burden of care in people with a major health condition that has a high personal and community cost. Heart, stroke and vascular diseases are collectively one of the leading causes of premature death and disability in the developed world. More than 75% of people with stroke have coronary heart disease and are 2–3 times more likely to be hospitalised with coronary heart disease and heart failure than the rest of the population [55], which contributes to high health costs. Low levels of physical activity are a known risk factor of coronary heart disease. After stroke, people have low levels of physical activity. This research will provide evidence of the effect of the application of a commercially available technology (treadmill training), used in a manner (high-intensity) that will enable stroke survivors to increase their fitness, thereby improving physical activity in the short-term. It is also anticipated that the self-management aspect of the intervention will result in the maintenance of high physical activity in the long-term, thereby improving quality of life and reducing the burden on others. Given the major demographic shift in developed nations involving significant growth in the aged population, this research could make an important evidence-based contribution to the promotion of healthy ageing.
